# *Lactobacillus paracasei* subsp. *paracasei* X12 Strain Induces Apoptosis in HT-29 Cells through Activation of the Mitochondrial Pathway

**DOI:** 10.3390/nu15092123

**Published:** 2023-04-28

**Authors:** Shumei Wang, Yi Shan, Shuang Zhang, Lanwei Zhang, Yuehua Jiao, Dijia Xue, Lili Zhang, Huaxi Yi

**Affiliations:** 1 College of Food Engineering, Heilongjiang Province Key Laboratory of Cold Region Wetland Ecology and Environment Research, Harbin University, Harbin 150086, China; smwang@hrbu.edu.cn (S.W.); shanyi_1@sina.com (Y.S.); 2 College of Food Science, Northeast Agricultural University, Harbin 150030, China; 3 College of Food Science and Engineering, Ocean University of China, Qingdao 266000, China; 4 Center of Drug Safety Evaluation, Heilongjiang University of Chinese Medicine, Harbin 150040, China; 5 College of Food Science and Engineering, Inner Mongolia Agricultural University, Hohhot 010018, China

**Keywords:** *L. paracasei* subsp. *paracasei* X12, whole peptidoglycan, HT-29 cells, apoptosis, mitochondrial pathway

## Abstract

*L. paracasei* subsp. *paracasei* X12 was obtained from traditional cheese produced in northwestern China. In this study, we showed that whole peptidoglycan (WPG), extracted from *L. paracasei* subsp. *paracasei* X12, inhibited proliferation and induced apoptosis in HT-29 cells in a dose-dependent manner. In addition, WPG-induced apoptosis was associated with the loss of mitochondrial membrane potential (Ψm), the release of cytochrome c (Cyto-C) from mitochondrialto cytosolic spaces, activation of Caspase 3, and accumulation of intracellular reactive oxygen species (ROS). Finally, semi-quantitative RT-PCR showed that these events were accompanied by upregulation of proapoptotic genes (Bax or Bad) and downregulation of antiapoptotic genes (Bcl-xl). Taken together, our results demonstrated that WPG induced apoptosis in HT-29 cells through activation of the mitochondrial pathway. WPG exerted only minor toxicity upon noncancerous cells and therefore might be used as a natural agent in the treatment of cancer in future.

## 1. Introduction

Colorectal cancer (CRC) is one of the most common cancers and one of the frequent causes of cancer deaths worldwide [1]. The human colon is a complex microbial ecosystem with several hundred bacterial species, some of which play essential roles in nutrient absorption and mucosal immunity and have been shown to protect against invasion by harmful substances and exert antimutagenic and anticarcinogenic properties [2]. The mechanisms by which bacterial species contribute to CRC are too complex to grasp completely, but increasing evidence displays the relation between the intestinal bacteria and CRC as well as dietary habits and inflammation, which are identified as playing a vital role in carcinogenesis [3].

Probiotics are nonpathogenic bacteria and possess the ability to beneficially influence host immune responses [4]. At present, probiotics have been confirmed to play protective functions against cancer development in animal models and reduce the incidence of postoperative inflammation in cancer patients [5]. Accordingly, some strains of probiotics have attracted a great deal of attention as they may be used as adjuvant agents for cancer prevention or treatment with minimal toxicity [6]. Among probiotics, the *lactobacillus* is one of the most generally utilized and thoroughly studied microorganisms [7]. Verma et al. [8] suggested that *L. rhamnosus* and *L. acidophilus* promoted apoptosis in colonic tumors in rats through descending Bcl-2 expression and enhancing the expression of wild-type P53. Shida et al. [9] demonstrated that cell wall fractions of *lactobacilli* significantly inhibited the growth of tumor cells in vitro via activating their innate immunity [10], and cell wall polysaccharides of *L. lactis* protected bacteria against phagocytosis by murine macrophages in vitro. Whole peptidoglycan (WPS) produced from the mutant of *L. casei shirota* secreted cytokines IL-6, IL-10, and IL12 after being co-incubated with murine macrophages. These results emphasized the function of WPS in immunosuppression [11].

Our previous research also showed that cell walls extracted from *L. paracasei* subsp*. paracasei* X12 exerted significant antiproliferative activity against HT-29 cells [12]. Gram-positive bacterium cell is encased in a cell wall characterized by a thick peptidoglycan sacculus that serves as a skeleton for the attachment of other components [13]. Peptidoglycan (PG), which makes up as much as 80% of the dry weight of the bacteria body [14] and 90% of the bacteria cell wall [15], is the main component of the Gram-positive cell wall. PG is a polysaccharide consisted of alternating *N*-acetylglucosamine (GlcNAc) and *N*-acetylmuramic acid (MurNAc) [16] that are cross linked via β-1,4 bonds. It exhibits various functions during bacterial growth, including preserving the intact bacterial cell structure and shape as well as reacting internal pressure [11].

Sarkar et al. [17] revealed that probiotic action of *bifidobacteria* was mediated by surface-associated structures such as exopolysaccharides or lipoteichoic acids. Accordingly, the anticarcinogenic activity of living *L. paracasei* subsp*. paracasei* X12 might be attributed to cell wall peptidoglycan. Previous studies have shown that existing anticancer therapies mostly focus on inducing apoptosis in cancer cells [18]. Apoptosis is induced by extracellular (extrinsic pathway, death-receptor-mediated) or intracellular (intrinsic pathway, mitochondria-mediated) signals [19]. The intrinsic pathway is a principal apoptotic pathway, elicited by mitochondria-mediated processes and induction of the caspase signaling cascade activation [20]. In turn, mitochondrial membrane potential (Ψm) dysfunction leads to the release of the proapoptotic molecule and cytochrome C (Cyto-C) from mitochondrial to the cytosolic spaces with the accumulation of reactive oxygen species (ROS) [21,22]. The release of Cyto-C and resultant over-production of ROS initiates (triggers) a caspase cascade, which is also regulated by the Bcl-2 family of proteins.

Findings from a number of reports have confirmed that fractions isolated from bacterial strains are capable of inducing apoptosis [23]. Sharma et al. [24] showed that the cyto-protective potential of *Enterococcus lactis* IITRHR1 and *Lactobacillus acidophilus* MTCC447 were involved with modulating the crucial end points of apoptosis as stimulated by oxidative stress. Findings generated from work within our laboratory have indicated that whole peptidoglycan (WPG) extracted from *L. paracasei* subsp*. paracasei* M5 exerted cytotoxic effects in HT-29 colon cancer cells as achieved by upregulating proapoptotic genes and downregulating antiapoptotic genes [25]. As *L. paracasei* subsp*. paracasei* X12 has been shown to be effective in inhibiting HT-29 cancer cell poliferation [12], we hypothesized that this anticarcinogenic activity of *L. paracasei* subsp*. paracasei* X12 was related to WPG. Therefore, the primary goal of this report was to examine whether anticarcinogenic activity of WPG contributed to activation of mitochondria-mediated pathway or the accumulation of intracellular ROS. To achieve this goal, a detailed analysis of the mechanisms involved with inducing the apoptotic signal pathway in response to WPG was performed.

## 2. Material and Methods

### 2.1. Lactobacillus Strain and Culture Conditions

*L. paracasei* subsp*. paracasei* X12 was obtained from Xinjiang traditional cheeses produced in northwestern China. This strain had previously been demonstrated to have good adhesion, antimicrobial activity [12,26], antioxidant activity [27], as well as antiproliferative activity on the HT-29 cancer cell line [12]. The strain was cultured in De Man, Rogosa and Sharpe (MRS) (Difco) broth with 0.05% (*w*/*w*) l-cysteine at 37 °C under anaerobic conditions and subcultured twice for 18 h before using and identified by 16S rRNA gene sequences.

### 2.2. Preparation of WPG from the Lactobacillus Strain

WPG was extracted from *L. paracasei* subsp*. paracasei* X12 by the method of [25]. Briefly, heat-killed cells of the X12 strain were treated with 0.5% Triton X-100 in 10 mmol L^−1^ 4-(2-hydroxyethyl)-1-piperazineethanesulfonic acid buffer (pH 7.0) for 1 h. The cells were washed successively with methanol:water (2:1, *v*/*v*), methanol, and acetone. The residue was incubated successively with Tris-HCl buffer (pH 7.2, containing 10 mmol L^−1^ MgCl_2_, 1 mg mL^−1^ trypsin, 100 µg mL^−1^ DNase and 100 µg mL^−1^ RNase), Tris-HCl buffer (pH 7.2, containing 0.5 mg mL^−1^ trypsin and 0.5 mg mL^−1^ α-chymotrypsin), 0.01 mol L^−1^ HCl (containing 1 mg mL^−1^ pepsin), Tris-HCl buffer (pH 7.4, containing 1 mg mL^−1^ pronase) at 37 °C with continuous shaking for 14 h. The residue was washed successively with methanol, methanol:chloroform (1:1, *v*/*v*), and chloroform and incubated 3 times with Tris-HCl buffer (pH 7.4, containing 1 mg mL^−1^ pronase) and then dialyzed against water for 3 days. The insoluble residue was treated with 5 mmol L^−1^ H_2_SO_4_ at 85 °C for 10 min. The residue was then centrifuged and dialyzed against water for 7 days and lyophilized. The protein content of WPG was determined by the Coomassie brilliant blue method.

### 2.3. Cell Lines and Cell Culture

HT-29 cells (human colorectal cancer cell line) were obtained from the Cancer Institute of the Chinese Academy of Medical Science (Beijing, China). HT-29 cells were cultured in 75-cm^2^ flasks containing complete RPMI-1640 medium (Hycolone, Logan, UT, USA) supplemented with 10% (*v*/*v*) inactivated (56 °C, 30 min) fetal bovine serum (Sijiqing, Hangzhou, China), with 1% (*v*/*v*) penicillin/streptomycin antibiotics (10,000 IU mL^−1^ and 10,000 µg mL^−1^; Gibco, Grand Island, NY, USA).

Vero cells (African green monkey kidney cell line) were obtained from the Harbin Veterinary Research Institute (Harbin, China). Vero cells were cultured in Dulbecco’s Modified Eagle medium (DMEM) supplemented with 10% (*v*/*v*) inactivated (56 °C, 30 min) fetal bovine serum (Gibco, USA). All cells were incubated in a CO_2_ incubator (HEPA class 100, Thermo Scientific, Waltham, MA, USA) at 37 °C with 5% CO_2_ and 95% filtered air in a humidified atmosphere. The medium was changed every 48 h.

### 2.4. Cell Viability Assay

The cell viability of cancerous cell line (HT-29 cell) and non-cancerous normal cell line (Vero cell) after treatment with different concentrations of WPG (10, 20, 40, 80, and 160 μg mL^−1^) was measured via MTT [(3-(4,5-dimethylthiazol-2-yl)-2,5-diphenyltetrazolium bromide)] assay and the trypan blue exclusion (TBE) assay. The cell treated with 7 μg mL^−1^ of 5-Fu was used as the positive control, and treated only with RPMI-1640 medium for HT-29 and DMEM for Vero were used as the negative control, respectively. TBE assay was performed as described previously by Nath et al. (2018) [28]. The numbers of dead and viable cells were counted under the light microscope (40-fold magnification). Result is presented as inhibition rate as calculated according to the following equation:

Inhibition rate = (apoptotic cell count/total cell count) × 100%

MTT assay was performed according to the method described by Wang et al. (2014) [12]. Absorbance was measured at 490 nm using an enzyme-linked immunosorbent assay plate reader (Bio-Rad-500, Hercules, CA, USA). Results were transformed into percentages based on the negative control. The inhibition rate was calculated according to the following formula:

Inhibition rate = [1 − (absorbance in test well)/(absorbance in control well)] × 100%

### 2.5. Analysis of Cell Apoptosis

Measurements of apoptosis and necrosis were performed as described by Sambrani et al. (2019) [29]. After incubation, the cells were washed with ice-cold phosphate-buffered saline (PBS, pH 7.2) and stained with 5 μL of Annexin V-FITC and 5 μL of propidium iodide (PI) at room temperature (25 °C) in the dark for 15 min. The cells were then analyzed by flow cytometry (FACS Calibur, Franklin Lake, NJ, USA) and the data were further analyzed by Cell Quest software. HT-29 cells that stained positive for Annexin VFITC and negative for PI were undergoing apoptosis. The cells that stained positive for both Annexin V-FITC and PI were either in the late stage of apoptosis or necrosis. The cells that stained negative for both Annexin V-FITC and PI were not undergoing apoptosis.

### 2.6. Morphological Observation of Apoptosis

Apoptotic cell morphology was detected using fluorescence and transmission electron microscopy. HT-29 monolayer, which was prepared on glass cover slips and placed in 6-well plates, was treated with WPG at 37 °C for 48 h. Hoechst fluorescent staining was performed by the method of Dhivya et al. (2016) [30]. After incubation, HT-29 monolayer was stained with 0.5 mL of Hoechst 33258 (Beyotime, Shanghai, China) for 5 min at 37 °C in the dark. Cells were then stained and imaged using fluorescence microscope (Olympus BX51, Manufacturer, Tokyo, Japan).

For transmission electron microscope, after incubation, HT-29 cells were fixed with 2.5% of glutaraldehyde for 48 h at 4 °C, followed fixed by 1% of osmium tetroxide fixation for 1 h at room temperature (25 °C). Cells were then dehydrated with successive treatments of acetone by the method of Di et al. (2017) [31]. Ultrathin sections were stained with uranyl acetate and lead citrate and observed with a Hitachi H-7650 transmission electron microscope (Hitachi Corp., Tokyo, Japan).

### 2.7. Measurement of Mitochondrial Membrane Potential and ROS Levels

HT-29 cells were seeded in 24-well plates with indicated concentrations of WPG. After incubation for 48 h, the cells were washed twice with ice-cold PBS (pH 7.2) and then stained with Rhodamine 123 (at the concentration of 200 µg mL^−1^ in PBS) [32] for mitochondrial membrane potential and 5 μM of DCFH-DA (an oxidant sensitive fluorescent probe dichlorofluorescein-diacetate) for ROS levels at room temperature for 30 min in the dark [33]. The stained cells were re-suspended in 300 μL of PBS and then measured by flow cytometry (FACS Calibur, Franklin Lake, NJ, USA). For mitochondrial membrane potential and ROS level, the excitation wavelengths were 507 nm and 485 nm, respectively, and the emission wavelengths were 532 nm and 535 nm, respectively. The data were further analyzed using Cell Quest Pro software.

### 2.8. Measurement of Cyto-Crelease

The amount of Cyto-C was detected according to the method described by Koul et al. (2017) [33]. HT-29 cells were seeded into 6-well plates and treated with WPG for 48 h. After treatment, the cytosolic and mitochondrial fractions were extracted from HT-29 cells and then were separated using a mitochondria isolation kit (C3601, Beyotime, China). Total protein of cytosolic and mitochondrial fractions was prepared on ice using cell extraction buffer (C2501, Haigene, Harbin, China) and protein samples were stored at −80 °C until use. The amount of Cyto-C in each of the cell extracts was quantified using an ELISA kit (Invitrogen, KHO1051, Carlsbad, CA, USA) according to the manufacturer’s instruction.

### 2.9. RNA Extraction and Semi-Quantitative RT-PCR

Total RNA was isolated using a total RNA extraction kit (BioFlux, BSC52M1, Tokyo, Japan) according to the manufacturer’s instruction. cDNA was generated with use of a reverse transcription kit (AE401, Transgen Biotech, Beijing, China) in accordance to the manufacturer’s instructions. Two microliters of the previous product was used for PCR using specific forward and reverse apoptotic gene primers (Table 1). The cycling conditions were as followings: initial denaturation at 94 °C for 2 min, followed by 35 amplification cycles at 94 °C for 15 s, annealing at 55 °C for 30 s, extension at 68 °C for 60 s, with a final extension at 68 °C for 5 min. PCR products were then separated by 1.5% agarose gel and stained with ethidium bromide [34]. Relative gene mRNA expression was analyzed by densitometry using image analysis software (Quantity One; Bio-Rad, Hercules, CA, USA). β-actin gene was used as a control, and its expression was considered as 100%.

### 2.10. Statistical Analysis

All experiments were performed in triplicate. Results were expressed as means ± SD. Statistical analyses were performed using SPSS 22.0 software. One-way ANOVAs with Duncan’s post hoc test were used for the data analysis. A probability level of *p* < 0.05 was used throughout this study.

## 3. Results

### 3.1. Effects of WPG on Viability of HT-29 Cells and Vero Cells

**Figure 1:**
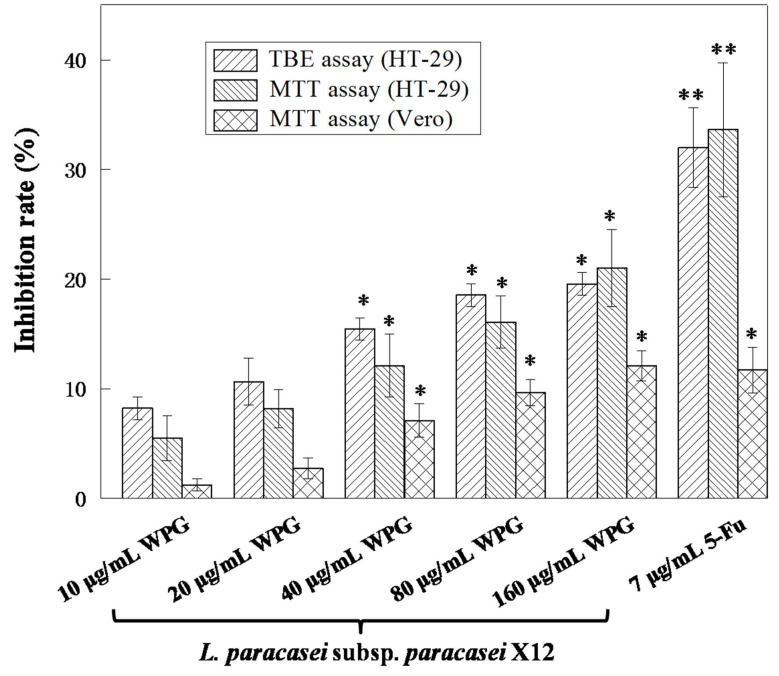
HT-29 and Vero cells were exposed to varying concentrations of WPG for 48 h. Inhibitory effects upon cell proliferation were determined via MTT and TBE assays. All values are presented as the mean ± standard deviation (SD) of three replications. Asterisks indicate samples that are significantly different from the control (* *p* < 0.05, ** *p* < 0.01).

The cytotoxicity of WPG was examined in cancer cell line HT-29 [35] and noncancerous cells line Vero [36] through MTT and TBE assays at 48 h. We had established previously that 10.31 μg mL^−1^ of 5-Fu produced an approximately 50% inhibitory rate on HT-29 cells (IC50) [25]. Therefore, 7 μg mL^−1^ of 5-Fu (a concentration lower than 10.31 μg mL^−1^) was used as a positive control [37] and HT-29 treated only with RPMI-1640 medium was used as the negative control within our experiments. Figure 1 illustrated the cell viability of both cell lines treated with increasing concentration of WPG. The cell viability of treated cells had been demonstrated as significant changes compared to the negative control cells. WPG exposure for 48 h significantly inhibited HT-29 cell growth in a concentration dependent manner, with a maximal inhibitory rate at a concentration of 160 μg mL^−1^ (*p* < 0.05). Nevertheless, at the lowest concentration of 10 μg mL^−1^, the inhibition rates just were 8.24 ± 1.03% (TBE) and 5.49 ± 2.03% (MTT), respectively. In response to treatment with the maximum concentration of 160 μg mL^−1^, the inhibition rates were 19.58 ± 1.03% (TBE) and 21.02 ± 3.54% (MTT), respectively. Among all groups, 80 and 160 μg mL^−1^ were without significant difference compared to the positive control; however, they showed significantly much higher (*p* < 0.05) antiproliferative activity compared to the groups of 10, 20, and 40 μg mL^−1^ and the negative control. No statistically significant difference in inhibitory rates was found between the MTT and TBE assays, with both showing similar dose-dependent antiproliferative activity after treatment by WPG. When WPG was exposed to Vero cells for 48 h, no significant difference in inhibitory rates was observed, with a maximal inhibitory rate of 12.11 ± 1.38% at a concentration of 160 μg mL^−1^ of WPG and with a inhibitory rate of 11.71 ± 2.07% at the positive control. Antiproliferative activity of WPG on the noncancerous Vero cells was significantly lower than that observed on cancerous HT-29 cells via MTT and TBE assays. These results demonstrated that WPG exerted a greater degree of sensitivity upon cancerous versus noncancerous cells.

### 3.2. WPG Induces Apoptosis in HT-29 Cells

**Figure 2:**
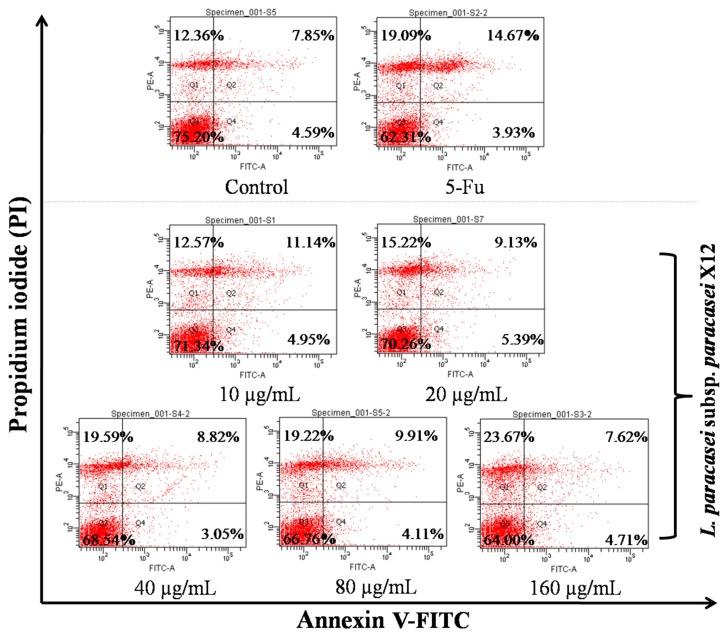
HT-29 cells were treated with WPG for 48 h and then stained with Annexin V-FITC/PI. HT-29 cells staining positive for Annexin V-FITC and negative for PI were showing initial stages of apoptosis. Cells staining positive for both Annexin V-FITC and PI were either in the terminal stages of apoptosis or already necrotic. Cells staining negative for both Annexin V-FITC and PI were viable with no indication of apoptosis. After staining, cells were subjected to flow cytometric analysis. The cells treated with 7 μg mL^−1^ of 5-Fu were used as the positive control, and those treated only with RPMI-1640 medium were used as the negative control; the following were all the same.

To explore the anticancer activity of WPG, the Annexin V-FITC staining method was performed to evaluate the cell apoptosis of HT-29 cells induced by WPG. We found that in the negative controls, 75.20% of the cells were viable, 4.59% in early apoptosis, 7.86% in late apoptosis, and 12.36% in necrosis (Figure 2). In cells treated with WPG (10–60 µg mL^−1^ of WPG), a 3.86–11.2% decrease in normal cells, a 0.96–3.28% increase in late apoptotic cells, and a 0.21–11.31% increase in necrotic cells were observed in comparison with the negative control. Specifically, when cells were treated with 80 and 160 µg mL^−1^ of WPG for 48 h, we observed 8.44% and 11.2% decreases in normal cells and 6.86% and 11.31% increases in necrotic cells, respectively, compared to that in the negative controls (*p* < 0.05). For the cells treated with the positive control (5-Fu), a 12.89% decrease in normal cells, a 6.81% increase in late apoptotic cells, and a 6.73% increase in necrotic cells were observed in comparison with the negative control. Significant induction of apoptosis was observed inHT-29 cells after treatment with WPG when compared with the negative control.

### 3.3. Effects of WPG on Morphology of HT-29 Cells

**Figure 3:**
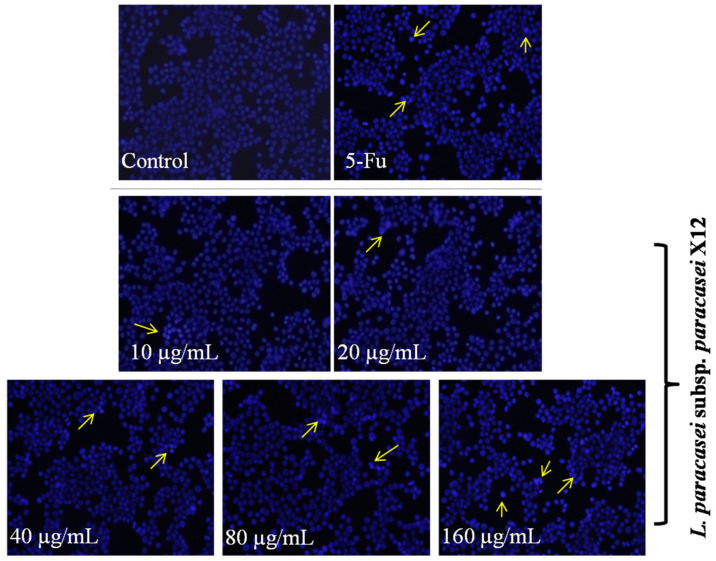
Apoptotic cells were evaluated with the fluorescence microscopy after Hoechst fluorescent staining. The cells that the yellow arrows point to are apoptotic cells.

Morphological observations on HT-29 cells were performed as a means to supplement these findings of apoptosis induced by WPG. Morphological characteristics of apoptotic cells were assessed by fluorescence and transmission electron microscopy. Hoechst 33258 staining was conducted to further confirm the apoptosis of HT-29 cells treated with WPG. As shown in Figure 3, the cells appeared circular or elliptical, without condensation of the nucleus being presented in negative control group and the group of 10 μg mL^−1^ of WPG. In contrast, the cells showed markedly condensed dots known as apoptotic bodies and morphological changes significantly in nuclei after treatment by 20, 40, 80, and 160 μg mL^−1^ of WPG. Furthermore, the cells exhibited the most obvious morphological changes treated with 80 and 160 μg mL^−1^ of WPG. Therefore, based on the results above, WPG indeed induced HT-29 colon cancer cell apoptosis, especially the high concentration groups of 80 and 160 μg mL^−1^.

**Figure 4:**
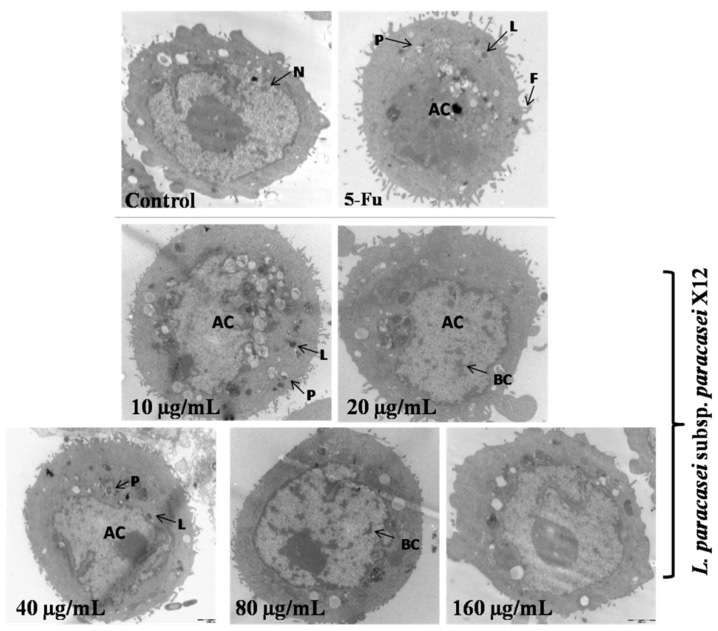
IEC-6 cells were investigated via transmission electron microscopic (original magnification 10,000×). AC: apoptotic cells; L: lysosomes; P: phagosome; F:filopodia; BC: broken chromatin; N: nucleus.

To investigate morphologic changes further in apoptotic cells, images were obtained via TEM with an original magnification of 10,000× (Figure 4). The morphology of the negative cells appeared normal, with a large single nucleus randomly distributed and organelles uniformly dispersed chromatin under transmission electron microscopy. Therefore, HT-29 cells treated with 40, 80, and 160 μg mL^−1^ of WPG began to show typical morphologic changes characteristic of apoptosis (Figure 4). Specifically, ultrastructural changes consisting of chromatin condensation, nuclear fragmentation, pseudopods, vacuoles, and apoptotic body formation were surrounding the nucleus and swollen mitochondria were present in apoptotic cells. These results were in agreement with those obtained from flow cytometric analysis.

### 3.4. Effect of WPG on Inducing Apoptosis via the Mitochondrial Pathway

**Figure 5:**
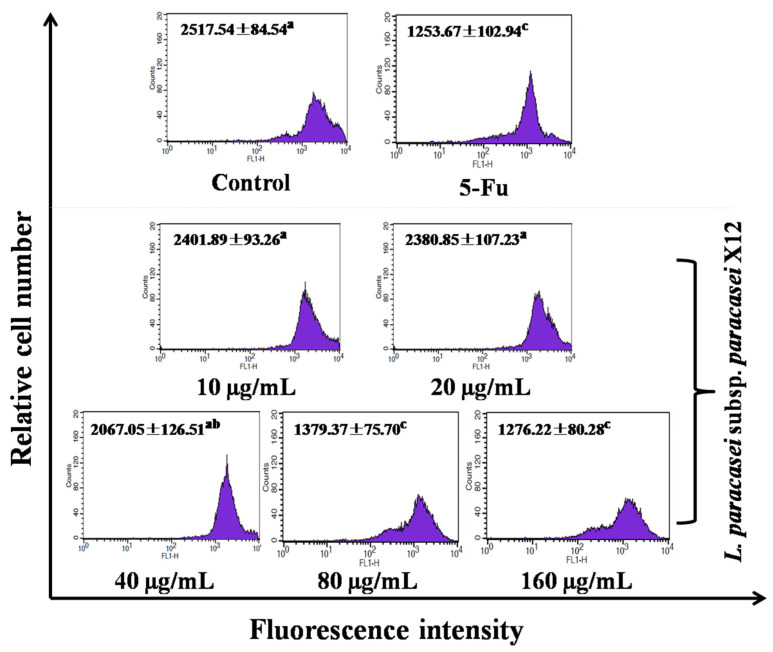
HT-29 cells treated with WPG for 48 h stained with Rhodamine 123, followed by analysis using flow cytometry. All values are presented as the mean ± standard deviation (SD) of three replications. (a–c) symbols indicate samples that are significantly different from the control (*p* ˂ 0.05).

In order to investigate whether mitochondria were involved in WPG induced apoptosis, we examined the transformation of the mitochondrial membrane potential (ΔΨm) in HT-29 cells via Rhodamine 123 staining. Mitochondria of normal cells absorb Rhodamine 123, and this absorbance declines when membrane potential decreases [38]. In this experiment, we found that 10, 20, 40, 80, and 160 μg mL^−1^ of WPG produced a breakdown of ΔΨm by 4.59%, 5.43%, 17.89%, 45.21%, and 49.31%, respectively, compared with untreated control cells (*p* < 0.01; Figure 5). The positive control of 5-Fu showed the highest breakdown of ΔΨm by 50.20%; however, there was no significant difference between the groups of 80 and 160 μg mL^−1^ of WPG.

**Figure 6:**
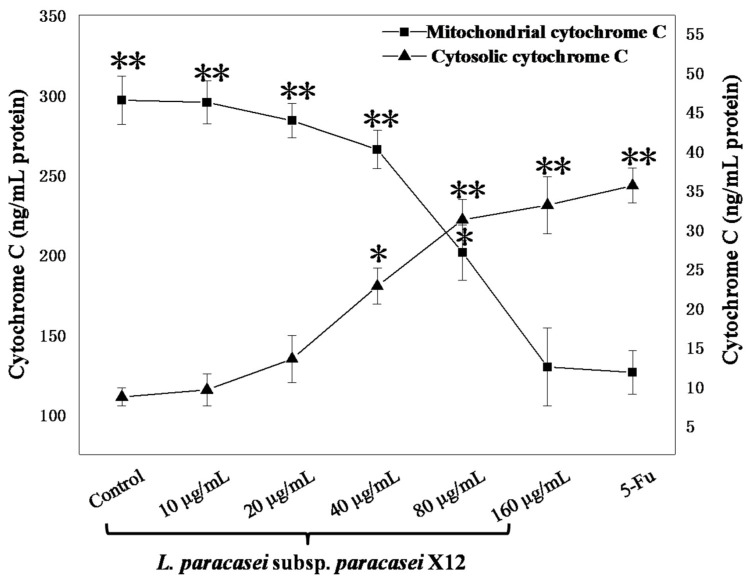
The effect of WPG on mitochondrial and cytosolic Cyto-C levels. Cyto-C was quantified using an ELISA. All values are presented as the mean ± standard deviation (SD) of three replications. Asterisks indicate samples that are significantly different from the control (* *p* < 0.05, ** *p* < 0.01).

A decrease in the mitochondrial membrane potential is also usually preceded or accompanied by Cyto-C release from the mitochondria into the cytosol. After exposure to 10, 20, 40, 80, and 160 μg mL^−1^ of WPG for 48 h, the amounts of Cyto-C in the cytosol of HT-29 cells increased by 10.52%, 55.84%, 163.12%, 260.81%, and 282.43%, respectively, compared with the untreated cells (Figure 6).However, the amounts of Cyto-C in the mitochondria decreased by 0.43%, 4.33%, 10.37%, 32.11%, and 56.28%, respectively, compared with the negative control (Figure 6). Therefore, the results showed that WPG exposure dose-dependently (10–160 µg mL^−1^) increased amounts of Cyto-C in the cytosol and decreased amounts in the mitochondria in HT-29 (*p* < 0.01).

### 3.5. Effects of WPG on Intracellular ROS

**Figure 7:**
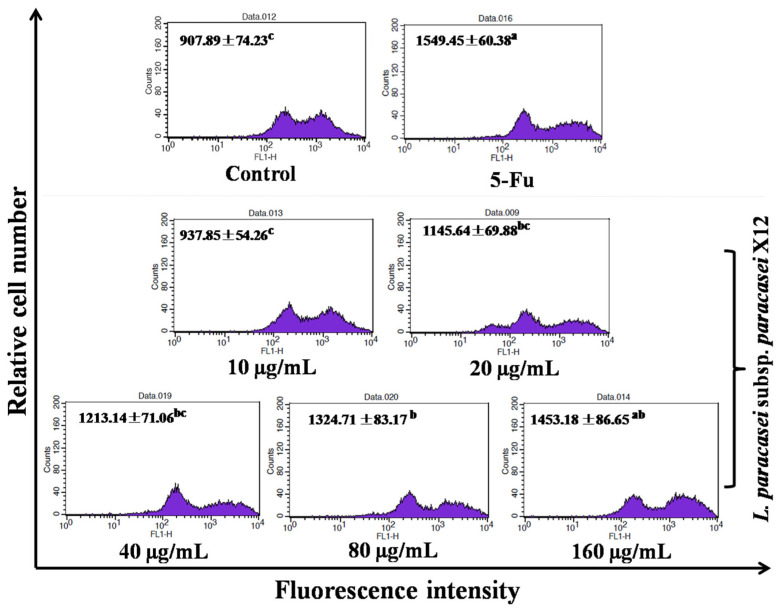
HT-29 cells treated with WPG for 48 h stained with DCFH-DA, followed by analysis using flow cytometry. All values are presented as the mean ± standard deviation (SD) of three replications. (a–c) symbols indicate samples that are significantly different from the control (*p*˂ 0.05).

Lastly, we examined whether intracellular ROS production was involved in WPG-induced apoptosis of HT-29 cells. As revealed with DCFH-DA staining, ROS levels were increased in WPG-treated versus the untreated control cells, with minimal levels observed in response to 10 μg mL^−1^ of WPG by 907.89 ± 74.23, without significant difference with the negative control. In contrast, with maximal level observed in response to 160 μg mL^−1^ by 1453.18 ± 86.65 (*p* < 0.01, Figure 7), a significant difference with the untreated control cells. When HT-29 cells were incubated with 10, 20, 40, 80, and 160 μg mL^−1^ of WPG for 48 h, we observed the increase in ROS level by 3.30%, 26.19%, 33.62%, 45.91%, and 60.06%, respectively, compared with that in the untreated cells. These results described above indicated that WPG-induced cell apoptosis was mediated by intracellular ROS generation in HT-29 cells.

### 3.6. Effects of WPG on Expression of Apoptosis-Related Genes

**Figure 8:**
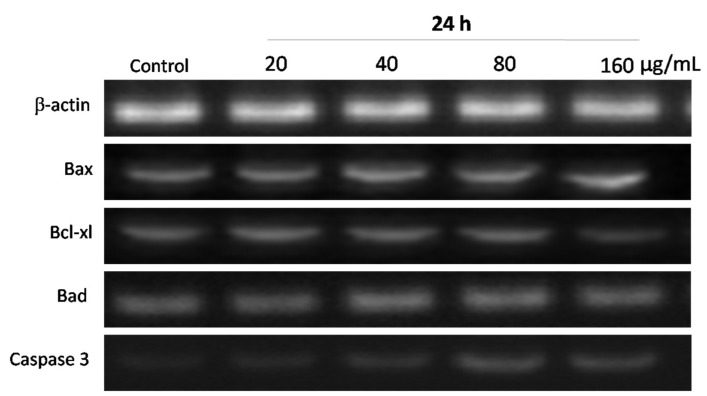
Expression of apoptotic signaling genes in HT-29 cells treated with WPG for 24 h as determined using semi-quantitative RT-PCR. Column 1: Control cells; Columns 2–5: WPG treated cells with 20 μg mL^−1^, 40 μg mL^−1^, 80 μg mL^−1^, or 160 μg mL^−1^, respectively.

**Figure 9:**
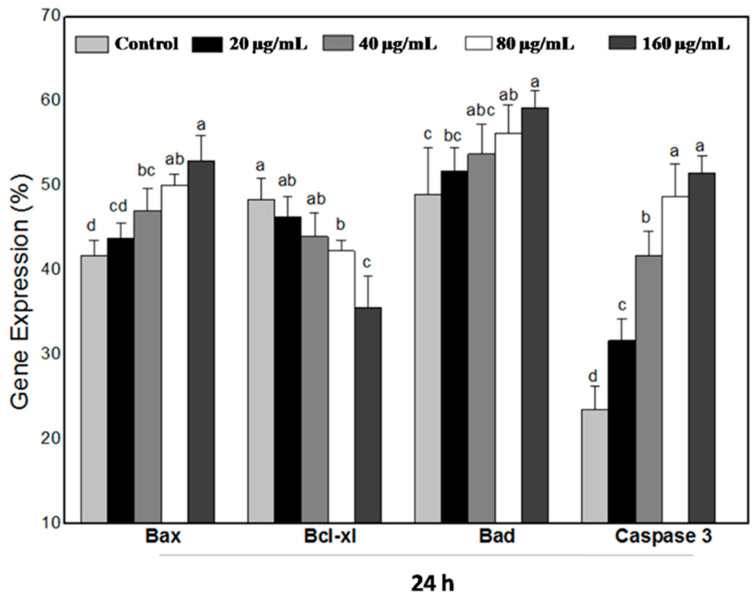
Results (HT-29 cells treated with WPG for 24 h) represent that of three separate experiments and quantitative expressions of apoptotic signaling genes (Bax, Bcl-xl, Bad, and Caspase 3) and are compared with that of the housekeeping gene β-actin (expression of β-actin was considered 100%). Symbols (a–d) indicate samples that are significantly different from the control (*p* ˂ 0.05).

The Bcl-2 (B-cell leukemia/lymphoma-2) protein family plays a vital role in regulating the mitochondria-dependent pathway of apoptosis. To examine the molecular mechanism associated with WPG induced apoptosis in HT-29 cells, the mRNA expression levels of Bcl-2 protein family members (including Bcl-xl, Bax and Bad) were examined via semi-quantitative RT-PCR. The housekeeping gene β-actin was used as a control. The forward and reverse primers of apoptotic genes are shown in Table 1. After exposure to WPG, mRNA expression levels of Bax and Bad in HT-29 cells were upregulated, whereas Bcl-xl was downregulated significantly. HT-29 cells were incubated with 20, 40, 80, and 160 µg mL^−1^ of WPG for 24 h, expression levels were increased by 4.79%, 12.64%, 19.92%, and 26.72% in Bax, and 5.57%, 9.61%, 14.58%, and 20.76% in Bad, respectively, whereas Bcl-xl expression levels were decreased by 4.10%, 9.00%, 12.53%, and 26.4% compared with the untreated cells (Figure 8 and Figure 9).

**Figure 10:**
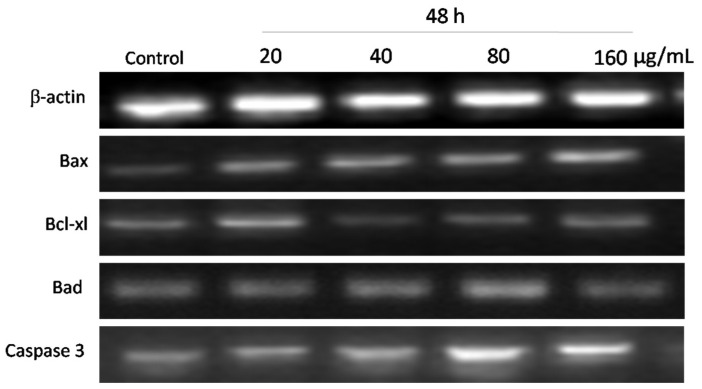
Expression of apoptotic signaling genes in HT-29 cells treated with WPG for 48 h as determined using semi-quantitative RT-PCR. Column 1: Control cells; Columns 2–5: WPG treated cells with 20 μg mL^−1^, 40 μg mL^−1^, 80 μg mL^−1^, or 160 μg mL^−1^, respectively.

**Figure 11:**
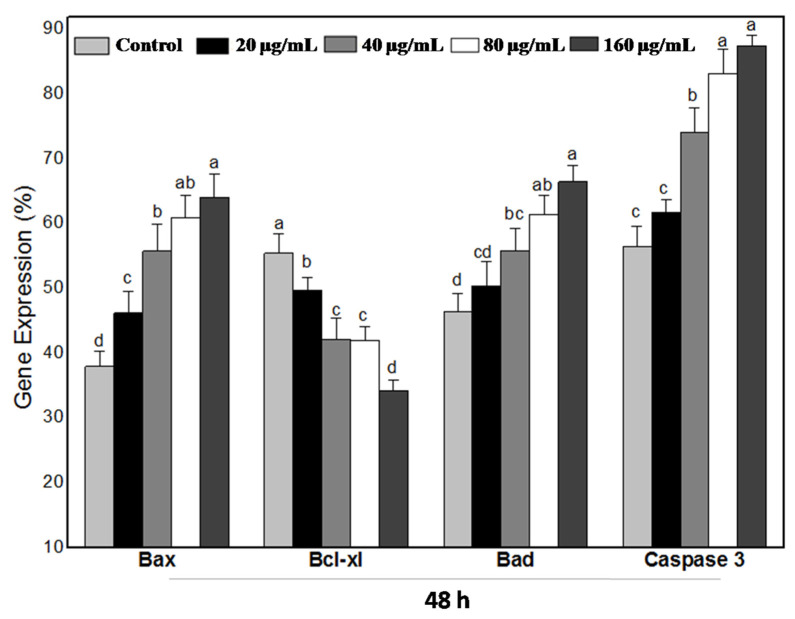
Results (HT-29 cells treated with WPG for 48 h) represent that of three separate experiments and quantitative expressions of apoptotic signaling genes (Bax, Bcl-xl, Bad, and Caspase 3) and are compared with that of the housekeeping gene β-actin (expression of β-actin was considered 100%). Symbols (a–d) indicate samples that are significantly different from the control (*p* ˂ 0.05).

Furthermore, when cells were treated with 20, 40, 80, and 160 µg mL^−1^ of WPG for 48 h, we observed the increase by 21.64%, 46.78%, 60.31%, and 68.60% in mRNA expression levels in Bax and the increase by 8.70%, 20.47%, 32.36%, and 43.24% in Bad and the decrease by 10.30%, 23.83%, 24.25%, and 38.14% in Bcl-xl, respectively, compared with the negative control (Figure 10 and Figure 11).

Apoptosis is executed by the coordinated actions of the caspase family. Therefore, we also examined Caspase 3 expression by semi-quantitative RT-PCR analysis. As shown in Figure 8 and Figure 10, treatment of HT-29 cells with WPG produced a significant activation of Caspase 3 as indicated by semi-quantitative RT-PCR. Expression levels of Caspase 3 in HT-29 cells incubated with 20, 40, 80, and 160 μg mL^−1^ of WPG for 24 h and 48 h were increased by 34.76%, 77.51%, 107.05%, and 118.87% and 9.36%, 31.40%, 47.54%, and 55.07%, respectively, compared with that in untreated cells (Figure 9 and Figure 11). Therefore, these findings showed that WPG induced apoptosis and was accompanied by a dose-dependent downregulation of antiapoptotic genes and upregulation of proapoptotic genes.

## 4. Discussion

In this study, we showed that WPG dose-dependently induced antiproliferative activity within HT-29 cells. These findings were in accord with related work, showing that assorted fractions from *lactobacillus* exerted anticarcinogenic effects, such as the cytoplasmic fraction and peptidoglycans from *lactic acid bacteria* (LAB) which inhibited proliferation of SNU-1 stomach adenocarcinoma cells [39]. Lipoteichoic acid, a major constituent of the cell wall of *lactobacillus plantarum*, was shown to exert antipathogenic effects and significant inhibitory effects on EHEC-induced apoptosis in intestinal epithelial cells of silvery pomfret [40]. Peptidoglycan (PG) derived from *Lactobacillus acidophilus* was selenized (Se-PG) via the HNO_3_-Na_2_SeO_3_ method and showed a great antitumor activity in HT-29 cells [41]. Peptidoglycan derived from *L*. *rhamnosus* MLGA induced the antimicrobial peptide defensin [42] derived from *L. casei* and decreased viability of various tumor cell lines [43].

Many of the medications used in the treatment of tumors were of limited utility due to toxic effects on noncancerous cells [44,45]. Therefore, the aim of cancer therapy is to increase the death or apoptosis of cancer cells without causing too much damage to noncancerous cells [46]. The WPG as extracted from *L. paracasei* subsp*. paracasei* X12 strain was shown to be safe on the noncancerous (Vero) cells used in this experiment as demonstrated in the MTT assay. Similarly, peptidoglycan fragments, isolated from *L. casei*, stimulated normal cells [47] and did not appear to affect the viability of noncancerous cells [43]; however, it impaired the entire metabolism of tumor cells and restored the apoptotic process [47]. The intracellular extracts and cell wall fractions of *lactic acid bacteria,* isolated from the feces of piglets, could prevent virus infection against Vero cells [48], and *lactic acid bacteria* isolated from cheese did not inhibit activity in Vero cells [49].

For this reason, WPG extracted from the *L. paracasei* subsp*. paracasei* X12 strain was manifested only minor toxic activity within Vero cells and might serve as an agent that can be used as a cancer treatment safely.

A decrease in cell proliferation or an increase in cell apoptosis represents an important feature in the treatment of cancer [46]. To examine the effect of WPG on HT-29 cell proliferation, the Annexin V-FITC staining assay was performed. Clear effects of WPG upon apoptosis were evident as observed in HT-29 cells treated with WPG. Such findings, indicating the importance of apoptosis in these antiproliferative effects in cancer cells, were supported by a number of related studies. Probiotics and their products showed the ability to inhibit cancer cell proliferation and induce cancer cell apoptosis [50]. For example, *L. rhamnosus* GG [51], *Lactobacillus plantarum* [52], and cytoplasmic fraction extracted from the *lactobacillus* strain [53] induced apoptosis in gastric cancer cells HGC-27, AGS cells, and SNU-1 cells, respectively, via modulation of signaling pathways. *Lactobacillus plantarum* possessed apoptotic induction in oral cancer KB cells through upregulation of PTEN and downregulation of MAPK signaling pathways [54]. Moreover, *Lactobacillus acidophilus* CICC 6074 [50] and *L. acidophilus* KLDS1.0901 [48] inhibited growth and induced apoptosisin colorectal cancer HT-29 cells and Caco-2 cells. As a result, Chen et al. [48] suggested that *L. acidophilus* KLDS1.0901 has the potential to develop a novel functional food for adjuvant treatment of colon cancer.

In general, apoptosis is characterized by a series of morphological changes, including chromatin condensation, nuclear fragmentation, DNA fragmentation and apoptotic body formation. In this study, cell shrinkage, chromatin condensation and morphological changes were observed in HT-29 cells after treatment with WPG as observed with optical, fluorescence, and transmission electron microscopy. These morphological characteristics of apoptotic cells were observed in Hoechst 33528 staining assays, which agreed well with the findings of transmission electron microscopy. Moreover, vacuoles and apoptotic body formation were present in these apoptotic cells as observed with transmission electron microscopy. In contrast, the negative control cells showed normal morphology with randomly distributed organelles, a single, large nucleolus, and uniformly dispersed chromatin. Accordingly, these results were in accord with the results obtained from flow cytometric analysis and are supported by a number of other studies. Fichera et al. [43] reported morphological alterations in bladder cells after exposure to *L. casei* and its derivative peptidoglycan. Moreover, Kim et al. (2004) [55] showed SNU-1 stomach adenocarcinoma cell chromatin condensation after exposure to the cytoplasmic fraction extracted from *lactobacillus,* and Choi et al. (2006) [45] showed characteristic morphological changes of HT-29 cells after exposure to the soluble polysaccharide fraction extracted from *L. acidophilus* 606.

One of the earliest events in the cell apoptosis cascade was the dissipation of △Ψm [56], and it was believed that ΔΨm might be an initiator of the mitochondrial apoptotic signaling pathway. A decrease in ΔΨm initiated the release of Cyto-C and other proteins from the mitochondria into the cytosol [56] and activated Caspase 9 and Caspase 3 [57]. Simultaneously, the decrease of ΔΨm and mitochondrial dysfunction was associated with modulatory effects exerted by the Bcl-2 family of proteins [22,58]. Probiotics have been shown to reduce mitochondrial membrane potential, which triggers the mitochondrial apoptosis pathway. *L. acidophilus* KLDS1.0901 reduced mitochondrial membrane potential and led to the apoptosis of HT-29 cells [59], *L. acidophilus* CICC 6074 caused the decrease of mitochondrial membrane potential in HT-29 cells, and induced the release of Cyto-C in the mitochondrial inner membrane into the cytosol [50].

In our study, involvement of the mitochondrial-mediated pathway in WPG-induced apoptosis was evaluated by examining a combination of parameters including the loss of ΔΨm, the release of Cyto-C, and the expression of Caspase 3 and Bcl-2 family proteins. Approximately 85% of total Cyto-C existed within the mitochondrial cristae, and the release of Cyto-C into the cytosol occurred via pores regulated by Bax and Bak proteins in the mitochondrial outer membrane [58,60]. Our results showed that ΔΨm began to decline after treatment with WPG, followed by an apoptosis cascade involving the release of Cyto-C from mitochondria into the cytosol and Procaspase-3 activation. Simultaneously, WPG downregulated Bcl-xl expressions and upregulated Bad and Bax expressions in a dose-dependent manner. These results confirmed that WPG induced apoptosis in HT-29 cells, at least in part, through activation of the mitochondrial damage-mediated caspase pathway.

Previous reports indicated that various forms of apoptosis were in part associated with inducing ROS formation [57], which produced a functional disorder of cell mitochondria resulting in apoptosis [61]. Probiotics can induce ROS production, the massively generated ROS aggravated the damage of mitochondria and apoptosis. Yue et al. [59] showed that *L. acidophilus* KLDS1.0901 induced ROS accumulation in HT-29 cells due to the apoptosis of the cells. To confirm whether ROS production was involved in WPG-induced apoptosis in HT-29 cells, ROS production was measured using DCFH-DA staining after treatment with WPG. The results showed that intracellular ROS levels were significantly increased in the WPG-treated cells compared with that in the untreated control cells. The increasing levels of intracellular ROS showed a dose-dependent relationship with the concentrations of WPG. These results demonstrated that WPG could produce an accumulation of intracellular ROS. We concluded that the WPG-induced apoptosis observed in these HT-29 cells involved the mitochondrial damage-mediated Caspase 3 pathway and was associated with an accumulation of intracellular ROS.

Wei et al. [61] suggested that apoptosis involved a programmed cell death in response to a series of morphological and biochemical changes. In this study, the results of WPG inducing apoptosis in HT-29 cells received from flow cytometry and morphological observation were consistent with those obtained from semi-quantitative RT-PCR analysis. The Bcl-2 family of proteins were recognized as important regulators of apoptosis [61,62] and usually were involved in cell apoptotic processes [63]. Bcl-xl were considered antiapoptotic proteins [61], with exerting antiapoptotic effects by blocking apoptosis through sequestration of proapoptotic Bcl-2 members [58]. Bax and Bad proteins were considered proapoptotic proteins [61]. Bad did not activate Bax; rather, it seemed to promote apoptosis by releasing Bid and Bim (a subset of Bcl-2 proteins) from Bcl-2 or Bcl-xl. Bid and Bim proteins activated Bax and Bak proteins which then permeabilized the outer membrane of the mitochondria leading to the release of Cyto-C from the inner-membrane space [50,58]. Our present findings showed that the apoptotic effects of WPG also worked through the Bcl-2 family of proteins, downregulating antiapoptotic genes (Bcl-xl) and upregulating proapoptotic genes (Bax, Bad).

Caspases also played a crucial role in the initiation and execution of apoptosis [56]. Both the extrinsic and intrinsic apoptosis signaling pathway involved and eventually converged upon the activation of the family of caspase. Different pathways are characterized with different caspases involved in the process of apoptosis. The intrinsic apoptosis pathway was often qualified by the activation of Caspase-3 and Caspase-9 [62] and the release of cytochrome c from mitochondria to cytoplasm [64]. Generally, cells underwent apoptosis through two major pathways, the death receptor-mediated pathway and the mitochondrial-mediated pathway, with both pathways finally converging through the activation of caspases [62]. Among these, Caspase 3 was an apoptotic executer and the activation of Caspase 3 was considered as an initiator of apoptosis [65]. Probiotic may play an important role in activation of the family of caspase and be consequently involved in inducing apoptosis of cancer cells. *L. acidophilus* CICC 6074 exerted anticancer effects via the activation of mitochondrial pathways by upregulating Bax, downregulating Bcl-2 and regulating Caspase-3 and Caspase-9 [50]. Heat-killed KU15176 exhibited a selective antiproliferative effect on AGS cell lines by regulating apoptosis-related genes such as Bax, Caspase-3, and Caspase-9 [62]. Lipoteichoic acid exerted significant inhibitory effects on EHEC-induced apoptosis by modulating the expression of Bcl-2, Bax and via inhibition of Caspase-3 and Caspase-9 activation [40]. In order to verify if the anticarcinogenic effects of WPG were linked to the Caspase 3, the expression of the Caspase 3 was assessed following WPG treatment as determined via RT-PCR assay. Our findings demonstrated that Caspase-3 was activated and therefore indicated that this cell’s apoptosis was Caspase-3-dependent.

## 5. Conclusions

In conclusion, our results demonstrated that WPG induced apoptosis in the human colon cancer cell line HT-29 in a dose-dependent manner. This capacity for WPG to induce of apoptosis involved activation of the mitochondria-mediated signal pathway and accumulation of intracellular ROS and was also controlled by the Bcl-2 family of proteins. This WPG, which was extracted from *L. paracasei* subsp*. paracasei* X12 strain, obtained from traditional cheese, exerted only minor toxic activity upon noncancerous cells. Therefore, WPG might serve as a natural agent for use in the treatment of cancer.

## Figures and Tables

**Table 1:** Primers used for apoptotic signaling genes.

Genes	Primers	
β-actin	Forward	TCACCCTGAAGTACCCCATC
	Reverse	CCATCTCTTGCTGCAAGTCC
Bax	Forward	TCCACCAAGAAGCTGAGCGA
	Reverse	GTCCAGCCCATGATGGTTCT
Bad	Forward	CCTTTAAGAAGGGACTTCCTCGCC
	Reverse	ACTTCCGATGGGACCAAGCCTTCC
Bcl-xl	Forward	ATGGCAGCAGTAAAGCAAGCGC
	Reverse	TTCTCCTGGTGGCAATGGCG
Caspase 3	Forward	TTTGTTTGTGTGCTTCTGAGCC
	Reverse	ATTCTGTTGCCACCTTTCGG

## Data Availability

Data sharing not applicable.
